# RPLP1, a Crucial Ribosomal Protein for Embryonic Development of the Nervous System

**DOI:** 10.1371/journal.pone.0099956

**Published:** 2014-06-24

**Authors:** Laura Perucho, Ana Artero-Castro, Sergi Guerrero, Santiago Ramón y Cajal, Matilde E. LLeonart, Zhao-Qi Wang

**Affiliations:** 1 Leibniz Institute for Age Research - Fritz Lipmann Institute (FLI), Jena, Germany; 2 Oncology and Pathology Group, Institut de Recerca Hospital Vall d'Hebron, Barcelona, Spain; 3 Faculty of Biology and Pharmacy, Friedrich Schiller University of Jena, Jena, Germany; University of Montréal and Hôpital Maisonneuve-Rosemont, Canada

## Abstract

Ribosomal proteins are pivotal to development and tissue homeostasis. RP Large P1 (*Rplp1*) overexpression is associated with tumorigenesis. However, the physiological function of *Rplp1* in mammalian development remains unknown. In this study, we disrupted *Rplp1* in the mouse germline and central nervous system (*Rplp1^CNS^* ^Δ^). *Rplp1* heterozygosity caused body size reductions, male infertility, systemic abnormalities in various tissues and a high frequency of early postnatal death. *Rplp1^CNS^* ^Δ^</emph> newborn mice exhibited perinatal lethality and brain atrophy with size reductions of the neocortex, midbrain and ganglionic eminence. The Rplp1 knockout neocortex exhibited progenitor cell proliferation arrest and apoptosis due to the dysregulation of key cell cycle and apoptosis regulators (cyclin A, cyclin E, p21^CIP1^, p27^KIP1^, p53). Similarly, *Rplp1* deletion in pMEFs led to proliferation arrest and premature senescence. Importantly, *Rplp1* deletion in primary mouse embryonic fibroblasts did not alter global protein synthesis, but did change the expression patterns of specific protein subsets involved in protein folding and the unfolded protein response, cell death, protein transport and signal transduction, among others. Altogether, we demonstrated that the translation “fine-tuning” exerted by Rplp1 is essential for embryonic and brain development and for proper cell proliferation.

## Introduction

The assurance of proper ribosome functionality is essential for the development of all multicellular organisms. Dysfunctions in most RPs induce developmental defects ranging from general translation impairment-related defects to tissue-specific phenotypes [1], [2]. More than 50 ribosomal protein (RP)-encoding loci were found to be mutated in a group of developmental abnormalities in *Drosophila* termed “minutes” [3]–[6]. “Minutes” are characterized by general developmental retardation, a reduced body size, short, thin bristles and diminished fertility, all of which are likely caused by a reduction in protein synthesis. In mammals, RP genes mutations are associated with tissue-specific abnormalities such as the mouse Tail-short (Ts), Tail-short shionogi (Tss) and Rabotorcido (Rbt) mutants, which present with skeletal abnormalities, short, kinky tails and neural tube defects such as exencephaly, spina bifida and cleft palate [7], [8]. Recent studies have shown that the developmental defects in Ts mutants are caused by mutations in *Rpl38*, thus affecting the production of RPL38 [1], which regulates axial-skeletal patterning by modulating the translation of a subset of Hox mRNAs [1]. Similarly, *Rpl24* hypomorphism causes Belly spot and tail (Bst) mutants, which present with white hind feet, kinky tails and ventral midline spots. *Rpl24* mutations impair *Rpl24* mRNA splicing and RPL24 production, thus affecting ribosome biogenesis, protein synthesis and the cell cycle [9].

The ribosomal stalk is a flexible lateral protuberance in the large ribosomal subunit that constitutes the specific elongation factor recognition motif [10]–[12]. In high eukaryotes, this stalk comprises RP Large P0 (P0) and two heterodimers formed by RP Large P1 (P1) and RP Large P2 (P2). This structure is known as the P complex or P proteins. P0 is connected to the rest of the ribosome through the ribosomal protein L12 and the 28S ribosomal RNA [13]. In contrast to other ribosomal proteins, P1 and P2 are not imported into the nucleus for their assembly, and constantly shuttle between the ribosome and the cytoplasm [14], [15]. Interestingly, P1 and P2 stabilize each other in the cytoplasm [16], [17]. Of importance is that ribosomal stalk protein alterations have been found in human tumors. For example, *Rplp1* mRNA levels are increased by five-fold in colorectal cancer tissues [18]. Similarly, human lymphoid cell lines containing mutant P53 were shown to overexpress P1 [19]. We previously found that the RNA and protein levels of P0, P1 and P2 (P proteins) were significantly increased in gynecologic tumors [20]. From a therapeutic point of view, gonadotropin-releasing hormone (GnRH) analogues, which are used to treat breast, prostate and ovarian cancers, have been found to exert their anti-proliferative effects through the downregulation of P1 and P2 [21], suggesting the potential clinical implications of targeting ribosomal stalk proteins. Interestingly, previous work from our laboratory showed that P1 overexpression allows cells to bypass replicative senescence, likely due to cyclin E overexpression consequent to increased E2F1 promoter activity [22]. Moreover, P1 was found to cooperate with Ras^Val12^ in the transformation of murine NIH3T3 cells [22].

The requirement of ribosomal stalk proteins in proliferating cells differs vastly among species. *S. cerevisiae* homologs of P1 and P2 are dispensable for viability [23]. In contrast, the depletion of P2 in human cells does not affect viability, but rather impairs proliferation, likely by affecting the efficiency of eukaryotic translation initiation factor 5B (IF-2)-mediated ribosome assembly [16], [24].

To date, the physiological function of *Rplp1* has remained elusive. This study explored the role of *Rplp1* in development and proliferation using *Rplp1*-deficient mouse models and derived cells. As proteins from the P complex are highly expressed in the fetal brain [25], we decided to explore the role of the P1 protein in brain development. We found that *Rplp1* was essential for embryonic and brain development. Rplp1 deletion induced proliferation defects and apoptosis *in vivo*. Moreover, Rplp1 deletion caused a senescence-associated proliferation arrest in primary mouse embryonic fibroblasts (pMEFs). Overall, we propose that *Rplp1* absence provokes a stress response associated with misfolded proteins that induces a different translation pattern, rather than a general disturbance of ribosomal function and/or protein synthesis.

## Materials and Methods

### Cell culture

Mouse embryonic fibroblasts (MEFs) were cultured in Dulbecco's Modified Eagle Medium (DMEM; Invitrogen Life Technologies, Carlsbad, CA, USA) supplemented with 10% fetal calf serum (FCS; Lonza, Basel, Switzerland), 2 mM L-glutamine (Invitrogen), 1 mM sodium pyruvate (Invitrogen, 11360-039), 100 units/mL of penicillin, 100 µg/mL of streptomycin (Pen/Strep; Invitrogen) and 0.5 mM β-mercaptoethanol (Invitrogen). Mouse embryonic stem (ES) cells (blastocysts derived from mouse strain 129/Sv) were cultured in Dulbecco's Modified Eagle Medium (DMEM) (Invitrogen) supplemented with 15% fetal calf serum (FCS) (Lonza), 2 mM L-glutamine (Invitrogen), 1 mM sodium pyruvate (Invitrogen), 1 mM MEM non-essential amino acids (Invitrogen), 100 units/mL of penicillin, 100 µg/mL of streptomycin (Pen/Strep; Invitrogen), 0.5 mM β-mercaptoethanol (Invitrogen) and 1000 U/mL LIF (Chemicon, Billerica, MA, USA). The culture medium was changed daily. Fresh medium was prepared every 2 to 3 days. All animal procedures followed Association for Assessment and Accreditation of Laboratory Animal Care guidelines and were approved by institutional Animal Care and Use Committee. Stable transfection was performed with 30 µg of retroviral vectors LMP^shRNA^ (control) LMP^shp53^, and LMP^shp16INK4A^ into Phoenix cells. Phoenix cells were maintained in a Minimum Essential Media (Gibco) supplemented with 50 µg/mL penicillin-streptomycin and 10% FBS. After 48 hours, the Phoenix supernatant was added into MEF cells, which were selected with 1 µg/mL puromycin and further treated with OHT during 4 days in order to induce the Rplp1 knock-down. For the transient transfection 2×10^5^ MEF cells were seeded per well in 6-well plates. After 24 h they were transfected with Lipofectamine 2000 with the p16^INK4AsiRNA^, p53^siRNA^ and Cy3 dye-labeled negative control (AM17010). Then, the cells were treated with OHT during four days in order to induce the Rplp1 knock-down. Stable cell lines were done according.

### Primary MEF (pMEF) immortalization

Early passage pMEFs (P2) were immortalized by p19^ARF^ knockdown. A plasmid encoding p19^ARF^ shRNA was transfected into the Phoenix E retroviral packaging cell line (based on HEK293 cells). After 15 hours, the medium was changed and the cells were incubated at 32°C to facilitate virus production. Forty-eight hours later, the viral supernatant was collected, filtered through a 0.45-µm filter and mixed 1∶1 with MEF medium and polybrene to a final concentration of 8 µg/ml. The mixture was incubated at RT for 5 minutes and then added to 30% confluent primary MEFs. The pMEFs were incubated at 32°C with 5% CO_2_. Twenty-four hours later, the medium was changed and the cells were transferred to a 37°C incubator. The cells were selected for 4 days post-infection with 2 µg/ml of puromycin.

### Growth curve

The growth curves were performed with freshly isolated pMEFs. Briefly, 1.5×10^5^ cells per clone were seeded in triplicates into 6-well plates. The triplicates were treated with 1 µM 4-hydroxytamoxifen (4-OHT) (Sigma-Aldrich, St. Louis, MO, USA) for 4 days, and the other triplicates were used as an untreated control. Every three days, the cells were trypsinized, counted and re-plated similarly. The growth curves were constructed by plotting the cumulative cell number versus the passage number.

### Proliferation assay

pMEFs were treated with 1 µM 4-OHT for 4 days. Three days later, the cells were incubated for 2 hours with 10 µM bromodeoxyuridine (BrdU). After incubation, the cells were washed with PBS 1X, harvested and fixed with ice-cold ethanol. Next, the cells were centrifuged at 1000×g for 10 minutes. The supernatants were aspirated and the pellets resuspended in 3 mL of 0.08% pepsin in 0.1 M HCl, followed by an incubation at 37°C for 20 minutes with occasional mixing. The cells were then centrifuged as described above, and the pellets were resuspended in 1.5 mL of 2 M HCl. The mixtures were incubated at 37°C for 20 min. Next, 3 mL of 0.1 M sodium borate were added, and the samples were centrifuged. The pellets were resuspended in 2 mL of IFA/Tween20 (0.5% Tween 20 in IFA) and centrifuged. The cells were subsequently stained with 75 µL of a FITC-conjugated anti-BrdU antibody (Becton Dickinson, Franklin Lakes, NJ, USA) at a 1∶5 dilution in IFA (10 mM HEPES, pH 7.4; 150 mM NaCl; 4% fetal bovine serum; 0.1% sodium azide) for 30 minutes on ice. Next, 2 mL of IFA/Tween 20 were added, and the cells were centrifuged and resuspended in 0.25 mL of IFA. Finally, 0.25 mL of a 20 µg/mL propidium iodide solution (PI) were added. The percentages of BrdU-positive cells were determined on a flow cytometer (FACSCanto, BD Biosciences; software FACS Diva, San Jose, CA, USA).

### Cell cycle

Confluent primary or immortalized MEFs were trypsinized and resuspended in 1 mL of PBS 1X and subsequently fixed with ice-cold absolute ethanol for at least one hour. The cells were then incubated with DNase-free RNaseA (100 µg/mL; Sigma-Aldrich) at 37°C for 30 minutes, after which 100 µL of 1 mg/mL propidium iodide (PI) (Sigma) were added. Finally, the cell cycle profiles were collected using a flow cytometer (FACSCanto, BD Biosciences; software, FACS Diva) (BD Biosciences).

### Apoptosis assay

Primary or immortalized MEFs were trypsinized and washed twice with PBS 1X. The supernatant was also collected to include the late apoptotic cells. The cells were resuspended in 0.1 mL of binding buffer (10 mM HEPES, pH 7.4; 140 mM NaCl; 2.5 mM CaCl_2_) containing Annexin V-FITC (1∶100 dilution; BD Biosciences). The mixture was incubated for 15 minutes at RT in the dark. Next, 400 µL of DAPI-containing binding buffer (final concentration, 0.2 µg/mL) were added. The samples were kept on ice for 15 minutes and then analyzed by flow cytometry.

### Senescence assay

Primary or immortalized MEFs growing in culture dishes or on coverslips were washed once with PBS 1X and fixed with 2% formaldehyde and 0.2% glutaraldehyde for 10 minutes at RT. Next, the cells were washed twice with PBS 1X and stained with a β-galactosidase staining solution (40 mM citric acid/sodium phosphate pH 6.0, 0.15 M NaCl, 2 mM MgCl_2_, 5 nM potassium ferrocyanide, 5 nM potassium ferricyanide, 1 mg/mL of X-Gal). The cells were incubated at 37°C in a dry incubator overnight. The reagents are included with the Senescence β-galactosidase staining kit (Cell Signaling, Danvers, MA, USA).

### Protein synthesis

To measure protein synthesis, we used a system based on the azide-alkyne reaction (Click-it Protein synthesis kit) (Invitrogen). Briefly, newly synthetized proteins are labeled with an azide (or alkyne)-coupled methionine analog. A fluorophore or HRP-conjugated alkyne (or azide) is then used for detection. In our study, we used a biotin-conjugated alkyne to perform western blotting. *Rplp1* ^F/F^; Cre-ER^T2^+ and *Rplp1* ^+/F^; Cre-ER^T2^+ immortalized MEFs were treated with 1 µM 4-OHT for 4 days or left untreated (control). Three days later, the cells were washed once with warm PBS and incubated for 1 hour with methionine-free DMEM (Invitrogen) to deplete the methionine reserves of the cells. Next, the medium was removed, and the cells were incubated for 3 hours in methionine-free DMEM with the methionine analog azide-homoalanine (AHA; Invitrogen). The cells were then washed once with PBS, followed by the addition of lysis buffer (1% SDS, 50 mM Tris-HCl pH 8, 1 mM Na_3_VO_4_, 10 mM NaF, 0.1 mM PMSF, 1X Roche Complete Protease Inhibitor Cocktail; Roche, Basel Switzerland). The cells were collected with a cell scraper and incubated for 30 minutes in lysis buffer. Next, the cell lysates were sonicated (5 30-second on/off cycles; BioruptorPlus; Diagenode, Seraing, Belgium) and centrifuged at 15,700×g for 15 minutes at 4°C. The supernatant was transferred to a fresh Eppendorf tube. A Bradford assay was used to measure the protein concentration as follows: 1 µL of protein extract was mixed with 999 µL of a 1∶5 dilution of Bradford solution (Bio-Rad) in water. The absorbance at 595 nm was determined with a spectrophotometer (Eppendorf BioPhotometer; Hamburg, Germany). Subsequently, 50 µg of protein were transferred to a new tube, to which were added 100 µL of biotin-alkyne in 2x reaction buffer (Invitrogen) and water to a final volume of 160 µL. The following reagents were then added sequentially: 10 µL of reagent C (CuSO_4_) (Invitrogen), 10 µL of reagent D (buffer additive 1) and 20 µL of reagent E (buffer additive 2). The mixture was shaken for 20 minutes, and then 600 µL of methanol, 150 µL of chloroform and 400 µL of water were added. The mixture was vortexed and centrifuged for 5 minutes at 13,200×g. The upper aqueous phase was discarded, and 450 µL of methanol were added. The protein solution was centrifuged as described above, and the methanol was discarded. The remaining protein pellet was washed again with methanol as described above, after which the pellet was air-dried. The pellet was resuspended in SDS-sample buffer 1X (see 2.6.2), vortexed for 10 minutes and then heated for 10 minutes at 70°C. A quarter of the mixture was separated by SDS-polyacrylamide gel electrophoresis and then transferred to a PVDF membrane (Bio-Rad). The membrane was blocked for 30 minutes with 5% BSA in TBS-T and then for 30 minutes with an ABC peroxidase reagent (Vectastain Burlingame, CA, USA), which reagent contains a biotinylated horseradish peroxidase (HRP) that had been pre-incubated with avidin at a specific ratio to form large complexes. The membrane was washed 3 times for 5 minutes each at RT. Finally, the membrane was developed with an ECL kit (Pierce, Appleton, WI, USA). Cells that had been treated with 1 µg/mL of cycloheximide (Sigma), which inhibits the elongation step of protein synthesis, were used as a negative control.

### Neurosphere formation assay

For this assay, freshly isolated neural stem cells were plated at a density of 8×10^5^ cells/T-25 flask in DMEM/F12 (Invitrogen) supplemented with 20 ng/mL of EGF (Peprotech, Rocky Hill, NJ, USA), 20 ng/mL of bFGF (Peprotech), 1X B-27 (Invitrogen), 100 units/mL of penicillin and 100 µg/mL of streptomycin (Pen/Strep; Invitrogen). The cells were incubated at 37°C with 5%CO_2_. Seven days later, the neurospheres were counted via microscopy to determine the number of neurospheres per mL.

### PCR

DNA was isolated from mouse tails or cells. For *Rplp1* PCR-genotyping, the following primers were used:

R1-1 5′-CGT GGT CTC CTA CTT CTG TG-3′

R1-2 5′-GAA AAG TGC CAG GAA ATC CAG T-3′

R1-15 5′-ATG CTG TGT CCA TTA TCC T-3′

The primers R1-1 and 2 yield bands of 145 bp for wild-type *Rplp1* and 201 bp for floxed *Rplp1*. If *Rplp1* is deleted, primers R1-1 and 15 yield a band of 411 bp. To detect the *Rplp1* targeted allele, the following primers were used:

R1-3 5′-CTT CAT GTA GAA AGT TTA GGA CTT G-3′

R1-4 5′-CTA GTG AGA CGT GCT ACT TC-3′

R1-5 5′-ATG TTT TGT AGT TCA GGC TGG-3′

Primers R1-3 and 4 yield a band of 444 bp for targeted *Rplp1* is targeted, whereas primers R1-3 and 5 yield a band of 158 bp for wild-type *Rplp1*.

For Cre PCR-genotyping, the following primers were used:

Cre1 5′-CGGTCGATGCAACGAGTGATG-3′

Cre2 5′-CCAGAGACGGAAATCCATCGC-3′

Actin-B2-1 5′-CACCGGAGAATGGGAAGCCGAA-3′

Actin-B2-2 5′-TCCACACAGATGGAGCGTCCAG-3′

The Cre1 and Cre2 primers detect the Cre transgene to yield a band of 643 bp. The Actin-B2-1 and Actin-B2-2 primers detect the Actin gene to yield a band of 294 bp and are used as a control. The PCR reaction conditions were as follows: For Rplp1, an initial denaturation step was performed for 3 minutes at 94°C, followed by 35 cycles of denaturation for 15 seconds at 94°C, primer annealing for 1 minute at 57°C and elongation for 2 minutes at 72°C and a final elongation step for 7 minutes at 72°C. For Cre, an initial denaturation step was performed for 3 minutes at 94°C, followed by 35 cycles of denaturation for 30 seconds at 94°C, primer annealing for 1 minute at 58.5°C and elongation for 2 minutes 30 seconds at 65°C and a final elongation step for 7 minutes at 65°C.

### Southern Blot

Genomic DNA from cells or mouse tissues was digested either with AseI for the short arm and PflFI for the long arm or HindIII. The digested samples were separated on a 0.8% agarose gel in TAE buffer (40 mM Tris Base, 20 mM acetic acid, 1 mM EDTA). The DNA was fragmented by depurination in 250 nM HCl for 20 minutes and denatured in 500 mM NaOH, 1.5 M NaCl for 30 minutes. The DNA was then transferred to a Hybond XL membrane (GE Healthcare Life Sciences, Cleveland, Ohio). Next, the membrane was incubated for 20 minutes in 40 mM NaPi and baked for 1 hour at 80°C, followed by DNA crosslinking (UV Stratalinker; Stratagene, La Jolla, CA, USA). The membrane was then prehybridized in Church's buffer (500 mM NaPi pH 8, 7% SDS, 1 mM EDTA) for one hour at 65°C. Subsequently, the membrane was hybridized overnight with a dCTP α-^32^P radio labeled probe in Church's buffer at 65°C. The sequences of the genomic probes were as follows:

**P3LA (for long arm):**

tcatttggctaggtggcaaggactgtgcctgcagattctccgtcactatcagttttataaattggagatgctcgcagttgctcatgctactatttcatatacatgaaaaaagatttgtttaaaaagtcgaatgtgctatttaacttggtacagctggcatttgactggaaaacaggtagttttttattataggatacaagctgatttcatagtgtagtcttcgattcttggagcgtttaagaaaagtttggagaaaaacattgctggtatattttgtggaaatgtgttttgaaatatagaaatctttactgcaagtgctcagaggtgctttcactgttgaggcacggtacatgtgaagtacctgttcctgtgttctacagggttctcctctaccagagtttcaacagcttgtactttttgatatggctgtatatgaagaatgttttctatttagatgcctcagacacacttaagcgttatattgatcctgctgatacttttaacagacctgctatttattacttcacaaagcagccataataattgggttttgatcaagaatgcgaaatggattatttgtcctgagctagccataaacaggaagacctagagtcacgacaactgtaccctttgccctttgttgcaatggaagctcccaaacagactatctcttcttcatgctgttgtccaaagatgcctgttggttgttgactaagaagagaacgttgaatgattttcatgtttagtgtgggcaggtttgatttcggtgaacttgcattgtaaactgctgcacttctaatggaacctcaaagtaatcctgggttgtgcttgtttcccaat.

**P1RA (for short arm):**

aggcctgttatcagtgatgggtgtccgtagtcttgattttgaagggtcttgcattaaggaaactgttctttttgtaaattgagatgtgccaattttcattaagcaccaagacagggacaggaccaatatctaaaccagaaataatggtcccatttgtgctgggtggtgggtgagaaacagtatcctggggttttatgggtgataacctgcactctgccggtcctgtctattcctgcatcttggatgttctaacatcttccgttgcagaaggctattttgcccagagaaaacagaagatgcttttaaattttttatttttaaatttttgaatcagggtgttcttctctatcccaagctggcctgaaactcactatgtagcctagagtggactggaaccttccagaaaccctcctgcctcagacttccaggtggctggtgtgacagctatactccacatgactgctttgtggaactttaggttctatgtccagctaagatattaaagctattaaaagcaaaggggcaaaaagaataaactattttgcagcttcctaggcccttccacagtgcattgaccttctcagagggtgcagacagtctccataccacctacatgcagggtccctgtttgaagagcaaactcagttccctatgtactggagccataggcctgatttggagggtcccaacaagtagagtctggccactttcctc.

**Probe G (for HindIII digestion):**

cagtgatagtattgtgaacctgggcttataatcttgtaacctgccacccacgtgtaggataatggacacagcatgcctggccataggctgttatttagctcaaatagttctgggcctccaggttctgctcaatttgacgtctacacttctgacatggaatgctaaggattgtattgcccaatgaaaatggatgttctggtccttgaggctcacctccttgatggggcattgtcttcccaaggtaaccttttgtggagttgggtgggggttagagtttaaggaagggtcttactgtgtagccttggctagtctggaaatcacctgcctttggaatttaacgaggggactggtaccctggttaattattgca.

The following day, the membrane was washed twice in washing buffer (1% SDS, 30 mM NaPi) for 5 minutes at 55°C, once for 15 minutes at 65°C and once for 30–45 minutes at 65°C. Finally, the membrane was exposed to a phosphoimager overnight and subsequently to X-Ray film for several days.

### Protein isolation

For protein isolation, the cells were trypsinized and washed twice with ice-cold PBS. After centrifugation at 240×g and supernatant removal, the cell pellet was resuspended in 50 L of RIPA buffer (50 mM Tris-HCl, 150 mM NaCl, 1 mM EDTA, 0.25% sodium deoxycholate, 1 mM Na_3_VO_4_, 10 mM NaF, 0.1 mM PMSF, 1% NP-40, 1X Roche Complete Protease Inhibitor Cocktail; Roche) and incubated for 30 minutes on ice. The lysate was then sonicated (30-second on/off cycles; Bioruptor Plus; Diagenode) and centrifuged at 15,700×g for 15 minutes at 4°C, followed by transfer to a fresh Eppendorf tube and storage at −80°C. A Bradford assay was used to measure the protein concentration as follows: 1 µL of protein extract was mixed with 999 µL of a 1∶5 dilution of Bradford solution (Bio-Rad) in water. The absorbance at 595 nm was determined with a spectrophotometer (Eppendorf BioPhotometer; Eppendorf). For protein isolation from mouse tissues, the tissues were previously snap-frozen in liquid nitrogen and stored at −80°C. A single tissue piece was minced with a sharp scalpel, followed by the addition of RIPA buffer. The tissue was subsequently homogenized with a tissue homogenizer (Polytron PT 2500E). The tissue lysate was incubated for 30 minutes on ice, followed by the above-described procedure.

### Western blot

For western blot analysis, the protein samples were separated by SDS-polyacrylamide gel electrophoresis (SDS-PAGE). After separation, the proteins were transferred to a PVDF membrane (Bio-Rad) that was subsequently blocked with 5% non-fat dried milk (NFDM) in TBS-T or in 5% BSA in TBS-T if antibodies against phosphorylated proteins were to be used. After blocking, the membrane was incubated overnight at 4°C with the primary antibody diluted in blocking solution. The membrane was then washed and incubated for 30 minutes to 2 hours at RT with the HRP-conjugated secondary antibody diluted in 5% NFDM in TBS-T. Finally, the membrane was washed and developed with an ECL kit (Pierce).

### Two-dimensional gel electrophoresis

Protein extracts from three different *Rplp1^i^* ^Δ^ pMEFs that had been treated with 4-OHT for four days or not (control) were separated by two-dimensional gel electrophoresis. The dysregulated proteins (p<0.01) in the *Rplp1^i^* ^Δ^ pMEFs were selected and identified by ESI-MS as described in [26].

### ROS assay

Control and inducible pMEFs were treated with OHT for 4 days. DCF-DA was then added to the cells for a 30 minutes incubation. The amount of DCF (the oxidized, fluorescent form of DCF-DA; Invitrogen) was then analyzed by flow cytometry.

### Isolation, fixation and embedding of mouse tissues

To isolate mouse or mouse embryonic tissues, the mouse was first sacrificed by cervical dislocation. After cleaning with 70% ethanol, the abdominal wall and peritoneum were opened to expose the organs or embryos, which were removed with forceps and sharp scissors, washed in PBS and fixed ON in 4% PFA at 4°C or in Roti-Histofix (4% formaldehyde, Roth) at RT. To isolate the brains from E18.5 embryos, the heads were removed and immersed in PBS in a Petri dish. The skulls were carefully removed with fine forceps. This procedure was performed under a stereomicroscope (Zeiss Stemi 2000; Zeiss, Oberkochen, Germany). After fixation, the tissues were washed in PBS and incubated for 30 minutes at 4°C in 30% ethanol, followed by a transfer to 50% ethanol, processing and paraffin embedding. The paraffin-embedded tissues were then cut into 5-µm-thick slices with a microtome (Microm, Cavriago, Italy). The slices were mounted on glass slides and dried overnight at 55°C. Hematoxylin and eosin (H&E) staining or immunohistochemistry (IHC) were then performed. All of the procedures performed in this study were approved by the Ethical Committee of Animal Research at the Leibniz Institute for Age Research.

### IHC and microscopy

To perform IHC analyses of the mouse embryonic brains, the paraffin sections were first deparaffinized. For antigen retrieval, the sections were incubated in 10 mM sodium citrate, pH 6.0 for 10 minutes at a sub-boiling temperature in a microwave. The sections were allowed to cool at RT for 30 minutes and were then washed three times with PBS for 5 minutes each at RT. Next, the sections were incubated in blocking solution (1% BSA, 5% goat serum, 0.4% Triton X-100 in PBS) to avoid non-specific antibody binding for 1 hour at RT in a humidified chamber. The blocking solution was then removed, and a primary antibody dilution in blocking solution was added. The sections were incubated overnight at 4°C in a humidified chamber with the primary antibody. Next, the sections were washed three times in PBS at RT and incubated with a fluorochrome-conjugated secondary antibody diluted in blocking solution. The sections were then washed three times in PBS for 5 minutes each and finally mounted with coverslips using a DAPI-containing mounting medium (ProLong gold; Invitrogen). The mounted sections were air-dried for several hours and stored temporarily at 4°C and long-term at -20°C. IHC images were acquired with a fluorescence microscope (Zeiss Axio Imager ApoTome or Zeiss Axio Imager M1; Zeiss) coupled to a charge-coupled device (CCD)-camera (Zeiss AxioCamMRm) and AxioVision software (Carl Zeiss). Bright-field images were obtained with a Zeiss Axio Imager M1 microscope fitted with a CCD-camera (Zeiss AxioCam MRc5). The number of cells positive for a certain marker was determined with the ImageJ software program.

### Terminal deoxynucleotidyl transferase dUTP nick-end labeling (TUNEL) of mouse brain sections

Paraffin sections of different embryonic brain stages were deparaffinized and incubated in 10 mM sodium citrate, pH 6.0 for 10 minutes at a sub-boiling temperature. The sections were cooled at RT for 30 minutes and washed three times for 5 minutes each at RT with PBS. The sections were incubated with a TUNEL reaction mixture (1x buffer, Amersham; 0.3 units/µL of terminal deoxynucleotidyltransferase, Amersham; 6.66 µM biotin-linked dUTP, Roche Diagnostics; water; Amersham, GE Healthcare Life Sciences) for 1 hour at 37°C in a humidified chamber. The sections were then washed three times for 5 minutes each in PBS at RT and incubated with a 1∶500 dilution of streptavidin-Cy3 (Sigma-Aldrich) in PBS with 1% BSA for one hour at RT. The sections were washed three times for five minutes each with PBS and mounted with coverslips, using a DAPI-containing mounting medium (ProLong gold; Invitrogen). The mounted sections were air-dried for several hours and stored temporarily at 4°C and long-term at −20°C.

### *In vivo* bromodeoxyuridine (BrdU)-proliferation assay

Pregnant females at E13.5 were injected intraperitoneally with a single 50-µg/g mouse body weight dose of BrdU (Sigma). After 30 minutes, the females were sacrificed by cervical dislocation and the embryos were collected. The embryonic heads were removed and fixed in 4% PFA at 4°C overnight. Portions of the embryos were used for DNA isolation and further analysis by PCR. The embryonic heads were processed as described in the IHC section. After deparaffination, the sections were incubated in 10 mM sodium citrate, pH 6.0 for 10 minutes at a sub-boiling temperature, cooled at RT for 30 minutes and washed three times for 5 minutes each with PBS at RT. Next, the sections were incubated in 2 M HCl at 37°C for 30 minutes to denature the DNA. The sections were washed three times in PBS for 5 minutes each, followed by a trypsin incubation (Zytomed, ZUCO43-15) for 20 minutes at 37°C. The sections were then washed three times for 5 minutes each in PBS and incubated for one hour at RT in a humidified chamber with blocking solution (1% BSA, 5% goat serum, 0.4% Triton X-100 in PBS). Finally, immunostaining was performed with an anti-BrdU antibody (Abcam, Cambridge, UK). The antibodies used are described in Table S1.

## Results

### Generation of *Rplp1* knockout mice

**Figure 1:**
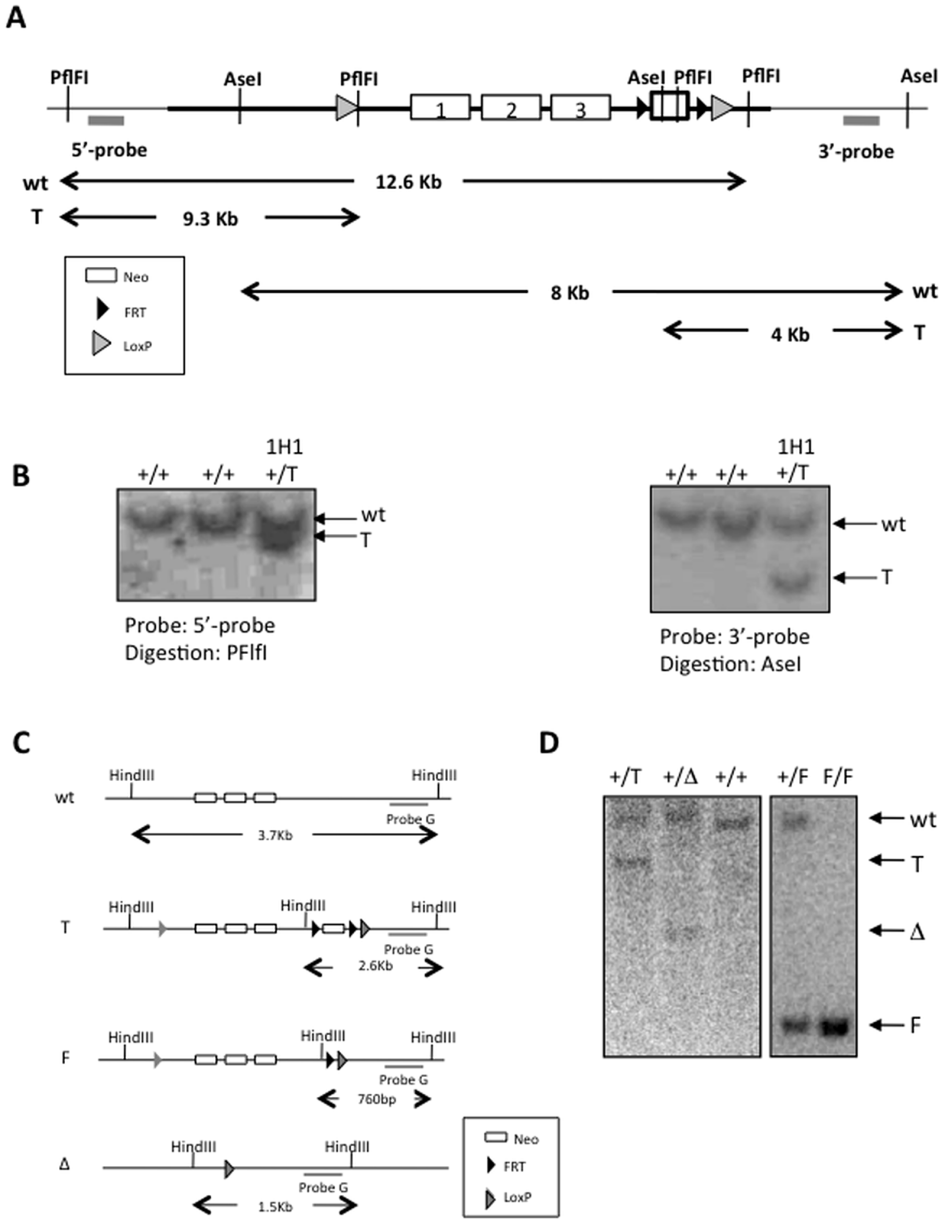
Generation of *Rplp1* knockout mice. (**A**) Gene targeting in ES cells. A Southern blotting strategy was used to screen the mutant alleles in ES cells. 5′- and 3′- external probes were hybridized to the Southern blots after genomic DNA digestion with *PflF*I and *Ase*I, respectively. (**B**) Southern blot analysis of gene-targeted *Rplp1* ES cells. Two strategies were used to confirm the correct integration of the gene-targeting vector, using the 5′- and 3′- probes indicated in (A). A *Rplp1* ^+/T^ clone (1H1) is shown. (**C**) Southern blotting strategy for *Rplp1* mutant alleles. (**D**) Southern blot of mouse tails, showing *Rplp1* ^+/+^, *Rplp1* ^+/T^, *Rplp1* ^+/F^, *Rplp1* ^F/F^ and *Rplp1* ^+/Δ^. Wt: wild-type allele. T: targeted allele. F: floxed allele. Δ: deleted allele.

To dissect the role of *Rplp1 in vivo*, we generated *Rplp1* constitutive and conditional knockout mice. Briefly, the two homologous regions of the Rplp1 gene were amplified by PCR from Sv129/J background mouse genomic DNA. These PCR fragments were subcloned into the DTA vector, which contains a neomycin (neo) resistance gene to ampicillin and the DTA (Diphtheria Toxin A) gene. A single LoxP site and anFRT-Neo-FRT-LoxP cassette were inserted by homologous recombination upstream of exon 1 and downstream of exon 3, respectively (Figure 1A). After gene targeting in embryonic stem (ES) cells, a southern blot analysis with 5′- and 3′-external probes confirmed the correct gene targeting event in the ES cells (Figure 1A and 1B). The targeted ES clone (1H1) was injected into blastocysts of C57BL/6N mice to generate chimeras that gave rise to germline offspring carrying the *Rplp1* targeted (T) allele. The germline offspring (*Rplp1* ^+/T^) was confirmed by southern blotting (Figure 1C and 1D). The intercrossing of *Rplp1* ^+/T^ mice with mice expressing Flp-recombinase (FLP) or Nestin-cre (NesCre) generated *Rplp1* floxed (*Rplp1* ^F/F^) mice or deleted *Rplp1* (*Rplp1* ^+/Δ^) mice, respectively (Figure 1C and 1D).

### *Rplp1* heterozygous mice exhibit severe developmental defects

**Figure 2:**
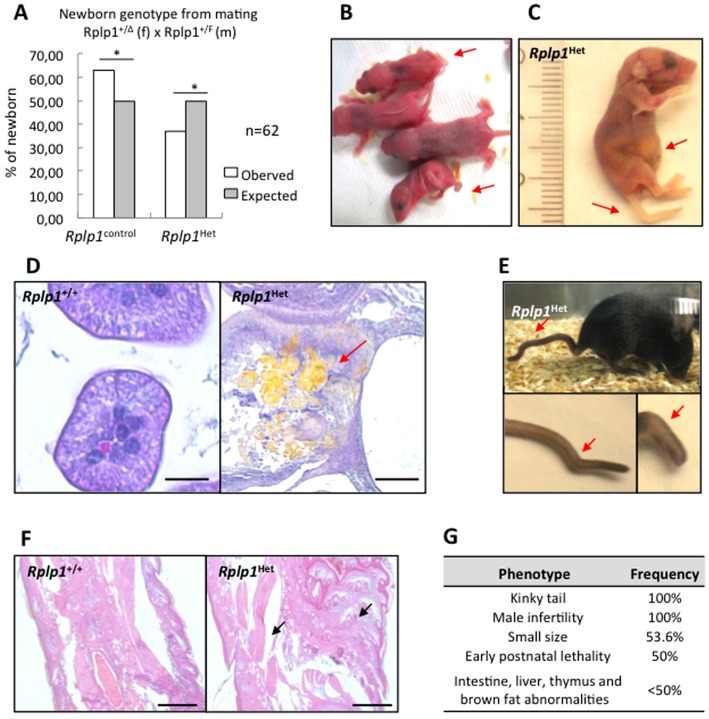
*Rplp1* ^Het^ mice exhibit developmental defects. (**A**) Offspring genotype distribution from matings between *Rplp1* ^+/Δ^ and *Rplp1* ^+/F^ mice. Pearson's chi-squared test was used for the statistical analysis. Χ^2^ = 0.04 (**B**) *Rplp1* ^Het^ mice (red arrows) are smaller than their littermates. (**C**) Detail of a *Rplp1* ^Het^ mouse with an abnormal, yellow-colored abdomen and a kinky tail. (**D**) H&E staining of *Rplp1* ^+/+^ and *Rplp1* ^Het^ mouse intestines from postnatal day 0. The intestines were perforated at different locations and meconium ileus (yellow) was present. Scale bar: 20 µm. (**E**) Kinky tails in *Rplp1* ^Het^ mice. (**F**) H&E staining of *Rplp1* ^Het^ and *Rplp1* ^+/+^ mouse tails. Scale bar: 500 µm. (**G**) Overview of the phenotypes observed in *Rplp1* ^Het^ mice.

We obtained *Rplp1* ^+/Δ^ and *Rplp1* ^F/Δ^ mice (*Rplp1* heterozygous mice, *Rplp1* ^Het^), which carry only one functional allele of *Rplp1*, from matings between *Rplp1* ^+/T^; NesCre^+^ mice and between *Rplp1* ^+/F^; NesCre^+^ mice. *Rplp1* ^Het^ mice were born at a significantly reduced frequency (Figure 2A). Moreover, approximately 50% of the *Rplp1* ^Het^ mice died during the early postnatal period. *Rplp1* ^Het^ newborn mice were smaller than the control littermates (Figure 2B) and exhibited a yellow abdominal coloration (Figure 2C). A histological analysis of the intestinal sections revealed perforations in the small and large bowel. Yellow-colored meconium ileus was present in the lesions. The epithelial structure was destroyed and inflammatory cells were present around these lesions (Figure 2D). Additional abnormalities were observed in the liver, where there was very little hematopoiesis and erythroblasts and granular cells were lacking (Figure S1). The thymuses in *Rplp1* ^Het^ mice were much smaller and exhibited abnormal organization. The brown fat adipoblasts were poorly differentiated (Figure S1). *Rplp1* ^Het^ mice exhibited rigid, shorter, kinky tails (Figure 2E); a histological analysis of *Rplp1* ^Het^ tail sections further revealed that the serial cartilage was disorganized and the long bones were abnormally enlarged (Figure 2F). Furthermore, *Rplp1* ^Het^ male mice were infertile, making the generation of *Rplp1* knockout mice impossible. However, the histological analyses of *Rplp1* ^Het^ testes sections revealed no differences between the *Rplp1* ^Het^ and control mice (Figure S1). The *Rplp1* ^Het^ mouse phenotype is summarized in Figure 2G.

### *Rplp1* conditional deletion in the CNS causes perinatal lethality and reduced brain size

**Figure 3:**
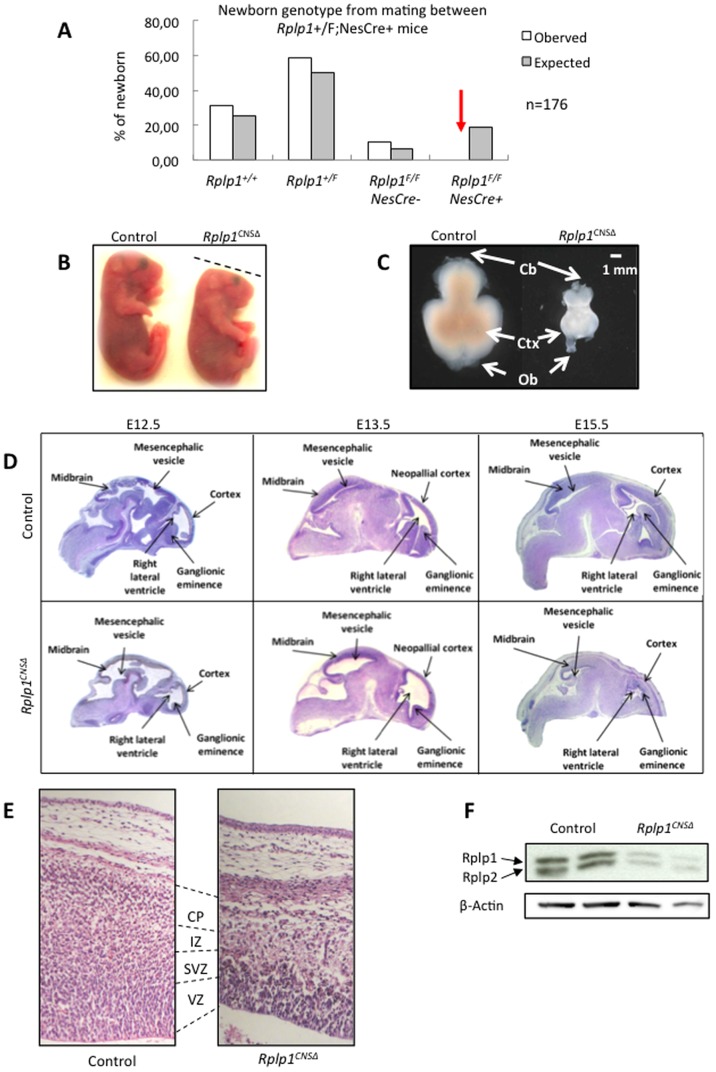
*Rplp1* ^CNSΔ^ mice die perinatally and have reduced brain sizes and morphological defects. (**A**) Table showing the genotypes of the mice obtained from matings between *Rplp1*+/F; NesCre+ mice. No *Rplp1* ^CNSΔ^ mice were obtained. (**B**) *Rplp1* ^CNSΔ^ and control E18.5 embryos. *Rplp1* ^CNSΔ^ E18.5 embryos exhibited abnormal head morphology. (**C**) Pictures of *Rplp1* ^CNSΔ^ and control E18.5 embryonic brains. The embryonic brain size is extremely reduced in *Rplp1* ^CNSΔ^ embryos. (**D**) H&E staining images of control and *Rplp1* ^CNSΔ^ embryonic brains at different stages. (**E**) Magnification of H&E stained control and *Rplp1* ^CNSΔ^ E15.5 neocortexes. (**F**) Western blot showing efficient *Rplp1* deletion in *Rplp1* ^CNSΔ^ mouse embryonic brains. RPLP2 expression was also downregulated in these mice. VZ: ventricular zone. SVZ: subventricular zone. IZ: intermediate zone. CP: cortical plate.

To study the role of *Rplp1* in the CNS, *Rplp1* conditional knockout mice (*Rplp1* ^CNSΔ^) were generated by crossing *Rplp1* floxed mice (*Rplp1^F^* ^/F^) with Nestin-Cre transgenic mice. Intercrossing between *Rplp1* ^+/F^; NesCre^+^ mice did not yield any *Rplp1* ^F/F^; NesCre^+^ (*Rplp1* ^CNSΔ^) offspring, indicating that *Rplp1* ^CNSΔ^ mice died during development or the early postnatal period (Figure 3A). We further analyzed the developmental stage at which the *Rplp1* ^CNSΔ^ mice died. We found viable *Rplp1* ^CNSΔ^ embryos at embryonic day (E) E12.5, E13.5, E15.5 and E17.5.

Furthermore, a cesarean procedure at E18.5 allowed us to recover viable *Rplp1^C^* ^NSΔ^ embryos. However, these were small, featured flat heads (Figure 3B) and had dramatically reduced brain sizes (Figure 3C). Overall, the mutant mice were unable to survive.

Because the brain defects at developmental stage E18.5 were dramatic, we analyzed the structure of the *Rplp1^CNSΔ^* embryonic brain at earlier stages. An histological examination revealed that at E12.5, compared with wild-type embryos, *Rplp1^CNSΔ^* embryos showed a reduced ganglionic eminence, a thinner neocortex and an enlarged right lateral ventricle (Figure 3D). At E13.5, the mutant embryos exhibited enlarged ventricles, atrophy of the neopallial cortex and roof of the midbrain, a reduced ganglionic eminence and reduced cellularity in the thalamus. At E15.5 the cortex and the midbrain were very small and atrophic, suggesting that brain growth was arrested at E13.5 (Figure 3D). The brain atrophy and arrested development explained the flattened shape of the head observed at later stages (e.g., E18.5; Figure 3B). At E15.5, the mutant embryos had a thinner cortex, which was associated with an abnormal cortical layer structure (Figure 3E). Histological analysis showed that the mutant cortex had a thinner ventricular zone (VZ) and was devoid of subventricular zone (SVZ) and cortical plate (CP). Overall, the cellularity was greatly reduced. The efficient deletion of *Rplp1* in *Rplp1^CNSΔ^* embryonic brains was already evident at E13.5 (Figure 3F). Notably, we found that P2 was also knocked-out in the *Rplp1^CNSΔ^* brains, possibly because P1 and P2 stabilize each other in the cytoplasm [17].

Taken together, our data show that *Rplp1* deletion in the CNS affected brain size and development, thus causing perinatal lethality (Figure 3D).

### *Rplp1* deletion in the CNS causes increased apoptosis and proliferation arrest in progenitor cells

**Figure 4:**
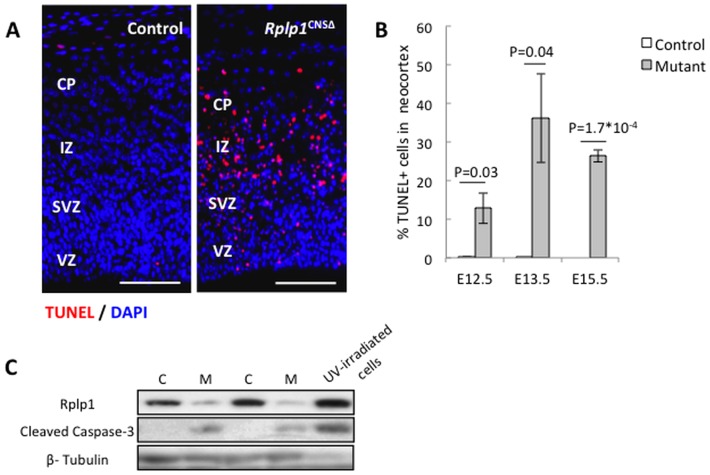
Increased apoptosis in the *Rplp1* ^CNS^ neocortex. (**A**) TUNEL staining of sagittal control and *Rplp1* ^CNSΔ^ embryonic brain sections. (**B**) Quantification of control and *Rplp1* ^CNSΔ^ embryonic brain TUNEL staining at E12.5, E13.5 and E15.5. (**C**) Western blot analysis of cleaved caspase-3 in E13.5 embryonic brain protein extracts. C: control. M: mutant. VZ: ventricular zone. SVZ: subventricular zone. IZ: intermediate zone. CP: cortical plate. Scale bars: 50 µm. N = 3. Error bars: SEM.

To study the causes of the decreased cellularity in the *Rplp1* ^CNSΔ^ neocortex, we evaluated apoptosis. Terminal deoxynucleotidyl transferase UTP nick-end labeling (TUNEL) staining revealed increased apoptosis in the neocortexes of the E12.5, E13.5 and E15.5 brain sections (Figure 4A and 4B). Concurrently, western blot analysis showed that caspase-3 activation was greatly increased in the E13.5 *Rplp1* ^CNSΔ^ brains, as determined by its cleavage (Figure 4C).

**Figure 5:**
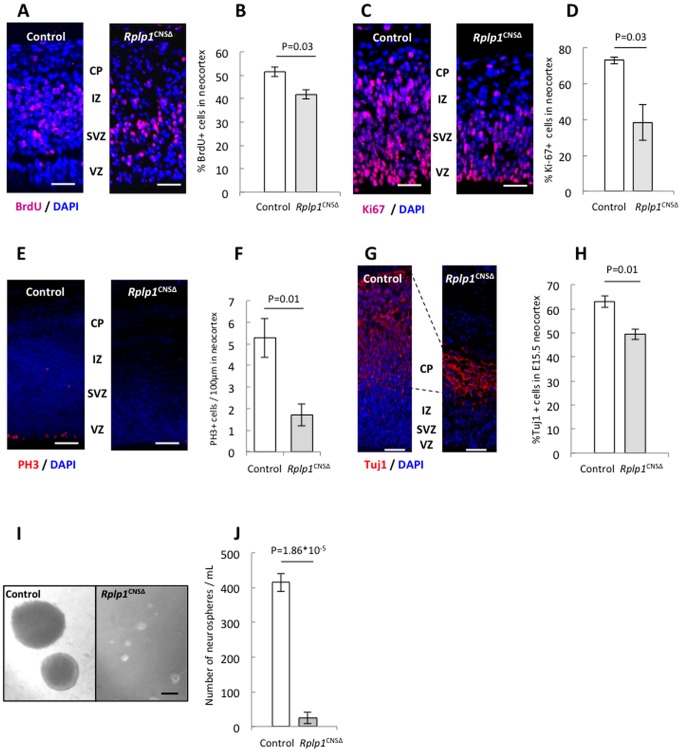
Proliferation defect in *Rplp1* ^CNSΔ^ mouse embryonic brains. (**A**) BrdU staining of control and *Rplp1* ^CNSΔ^ E13.5 embryonic brain paraffin sections after BrdU pulse-labeling for 1 h. Scale bars: 50 µm. N = 3. (**B**) Quantification of BrdU+ cells in the neocortex. (**C**) Ki67 staining in control and *Rplp1* ^CNSΔ^ E13.5 embryonic brain paraffin sections. Scale bars: 50 µm. N = 3. (**D**) Quantification of Ki67+ cells in the neocortex. (**E**) Paraffin sections of control and *Rplp1* ^CNSΔ^ E13.5 embryonic brains were stained for PH 3. Scale bars: 100 µm. N = 4. (**F**) Quantification of PH 3+ cells in the neocortex. (**G**) Paraffin sections of control and *Rplp1* ^CNSΔ^ E15.5 embryonic brains were stained for Tuj1. Scale bars: 50 µm. N = 3. (**H**) Quantification of Tuj1+ cells in the neocortex. (**I**) Control and *Rplp1* ^CNSΔ^ neurospheres formed *in vitro* after 7 days in culture. (**J**) Quantification of neurospheres per mL. Scale bars: 50 µm. N = 3. VZ: ventricular zone. SVZ: subventricular zone. IZ: intermediate zone. CP: cortical plate. Error bars: SEM.

Next, we examined whether *Rplp1* deletion caused a proliferation defect in the mouse embryonic brain. First, we performed an *in vivo* BrdU labeling assay by pulse-labeling the E13.5 embryos with BrdU for 30 minutes. We found that the number of BrdU^+^ cells was significantly reduced in the *Rplp1* ^CNSΔ^ embryonic brain, thus indicating a proliferation defect (Figure 5A and 5B).

Next, immunostaining of the E13.5 brain sections with an anti-Ki67 antibody revealed that the number of Ki67^+^ cells was significantly reduced in the *Rplp1^CNS^* ^Δ^ embryonic brain, compared with the controls (Figure 5C and 5D), suggesting the presence of fewer cycling cells. Staining of the *Rplp1* ^CNSΔ^ embryonic brain sections with an antibody against phosphorylated histone-3 (PH 3) consistently revealed reduced mitosis in the *Rplp1* ^CNSΔ^ neocortex (Figure 5E and 5F). To further analyze the proliferation and apoptosis of *Rplp1* ^CNSΔ^ neural progenitors, we performed a primary neurosphere formation assay and found that the *Rplp1* ^CNSΔ^ neural progenitors formed very few neurospheres and the sizes of the formed neurospheres were dramatically reduced (Figure 5I and 5J), indicating compromised proliferation and enhanced apoptosis.

Consequent to the reduced proliferation and increased apoptosis, postmitotic neurons (Tuj1+) were found to be greatly reduced in the mutant neocortex (Figure 5G and 5H). These observations well explain the absence of the CP and intermediate zone (IZ) layers (Figure 3E) and contribute to an explanation of the brain atrophy of *Rplp1* ^CNSΔ^ mice.

**Figure 6:**
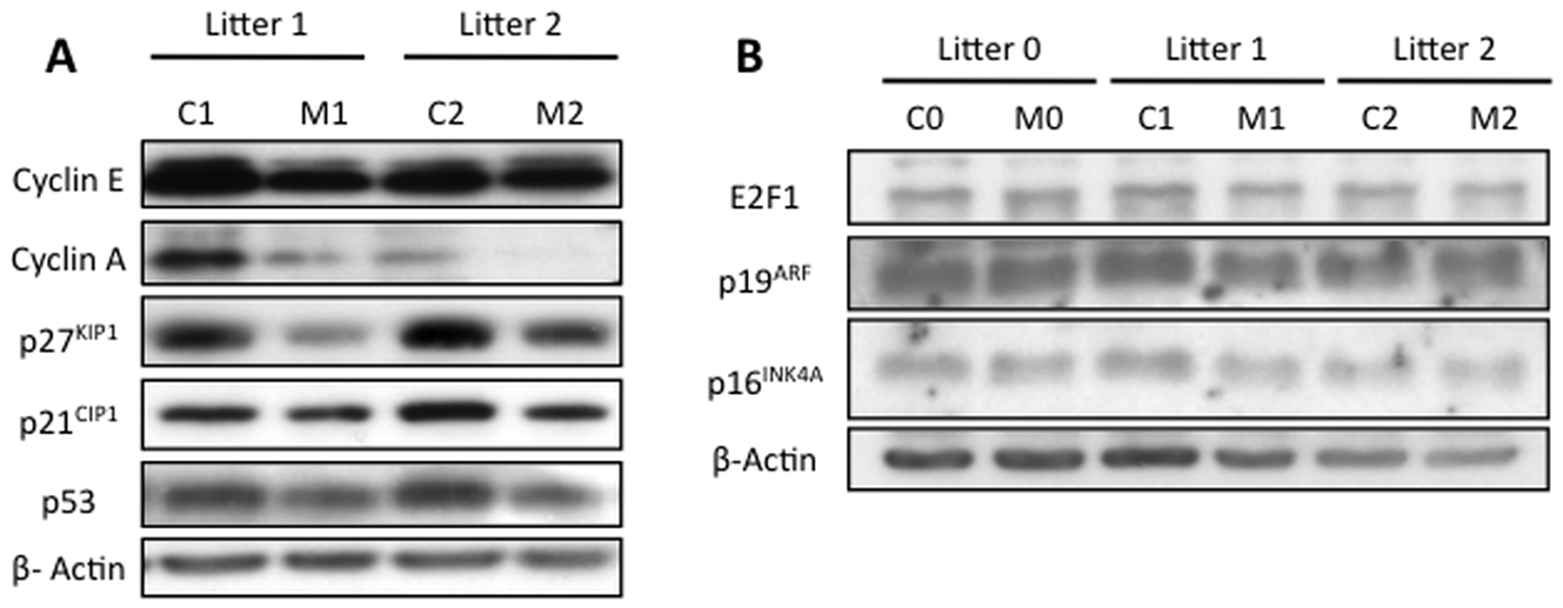
Cyclin A, cyclin E, P21^CIP1^, P27^KIP1^ and P53 expression are downregulated in *Rplp1* ^CNSΔ^ E13.5 embryonic brains. Western blot analysis of E13.5 embryonic brain samples was performed.

To further understand the proliferation defects observed in the *Rplp1* ^CNSΔ^ embryonic brains, the key cell cycle regulators in the *Rplp1* ^CNSΔ^ E13.5 brain were analyzed. The cyclin A and cyclin E protein levels were reduced in the mutant brain (Figure 6). Moreover, the protein levels of the CDK inhibitors p21^CIP1^ and p27^KIP1^ were reduced in the mutant brain (Figure 6). Similarly, p53 expression was slightly downregulated in the *Rplp1*-deleted embryonic brain (Figure 6) suggesting that p21^CIP1^ and p27^KIP1^ inhibition can be responsible for the apoptosis observed in the brain [27], [28]. We conclude that *Rplp1* deletion in the developing CNS caused perinatal lethality, reduced brain size and morphological brain defects consequent to greatly increased apoptosis in association with a proliferation defect.

### *Rplp1* deletion in MEFs causes a p16^INK4A^/pRb pathway-mediated proliferation defect and increased senescence

**Figure 7:**
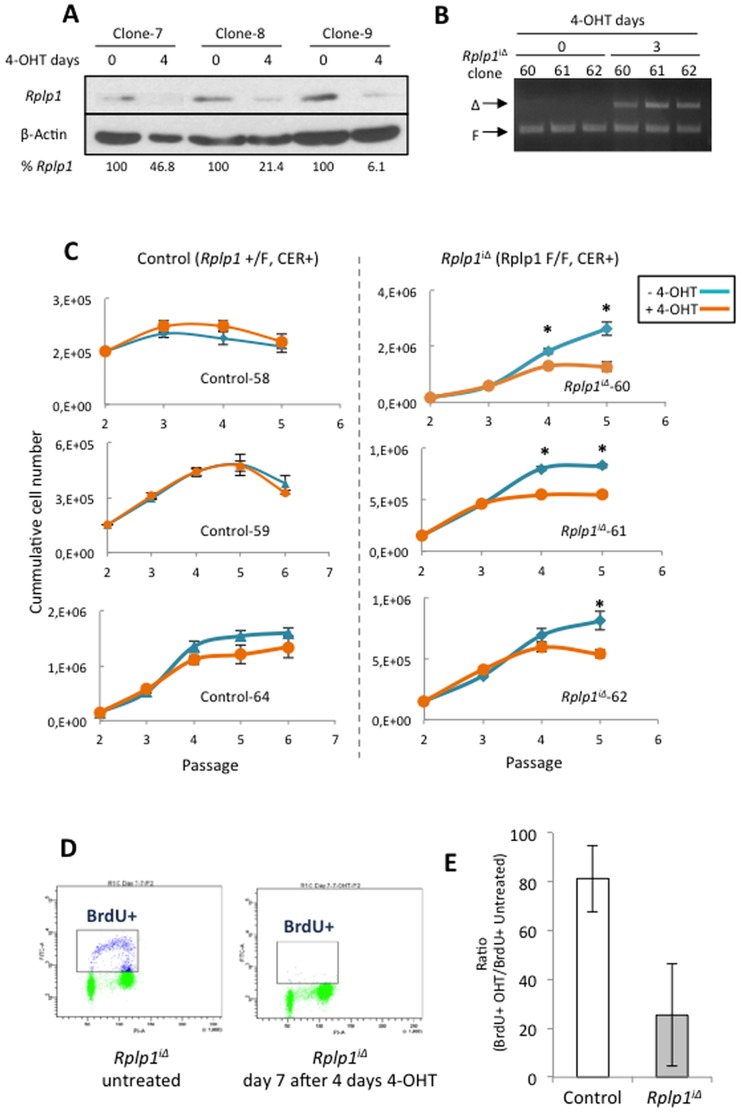
*Rplp1*-deleted primary MEF exhibit a proliferation defect. (**A**) Western blot analysis of *Rplp1* ^iΔ^ pMEFs that were untreated or treated for 4 days with 1 µM 4-OHT. (**B**) PCR, showing *Rplp1* deletion in the inducible MEF cell lines used in (c). (**C**) Growth curves of control (*Rplp1* ^+/F^ CER^+^) and *Rplp1*-inducible pMEF (*Rplp1* ^iΔ^), that were untreated or treated with 1 µM 4-OHT for 4 days. Student's t-test was used to perform the statistical analysis. *: P<0.05. (**D**) *Rplp1* ^iΔ^ and control pMEFs were treated with 1 µM 4-OHT for 4 days or left untreated. After 3 days, they were labeled with 10 µM BrdU for 2 hours, fixed and stained with an anti-BrdU antibody and PI. The cells were analyzed by flow cytometry. (**E**) BrdU^+^ cells were quantified, and the ratio of BrdU^+^ cells (treated/untreated) was plotted. Error bars: SEM.

**Figure 8:**
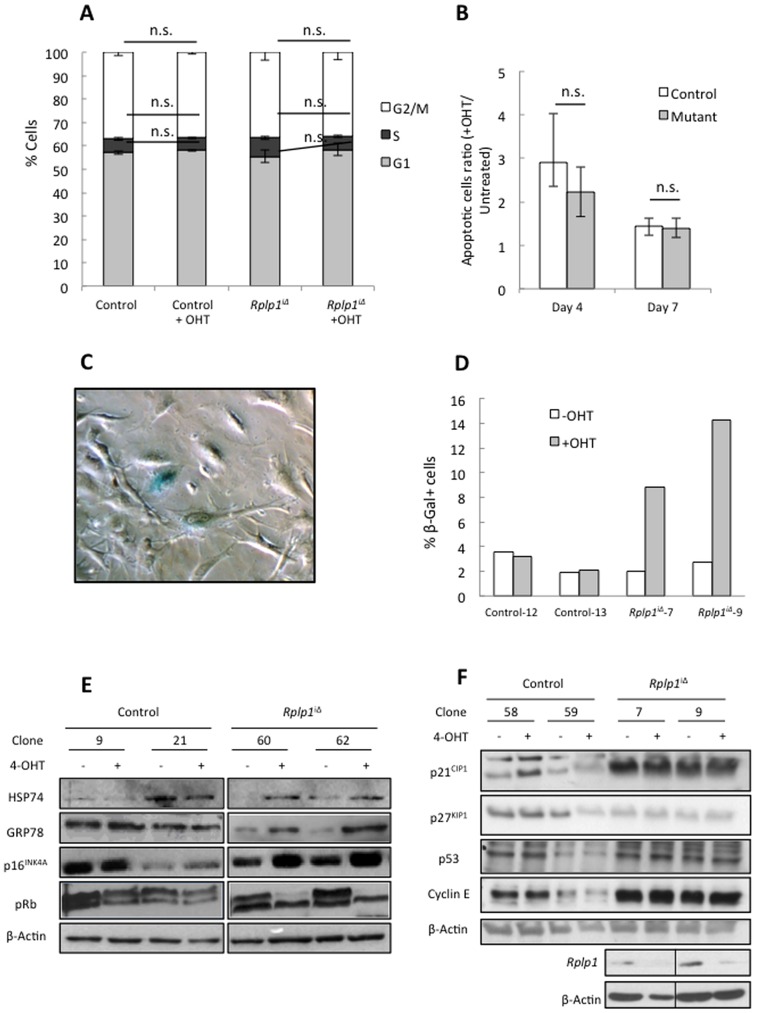
Increased senescence in *Rplp1*-deleted primary MEFs is mediated by the p16^INK4A^/pRb pathway. (**A**) Quantification of the cell cycle profiles of control and *Rplp1^i^* ^Δ^ pMEFs that were untreated or treated for 4 days with 4-OHT. Student's t-test was applied for the statistical analysis. n.s.: not significant. Error bars: SEM. N = 3. (**B**) *Rplp1* ^iΔ^ and control pMEFs were treated with 1 µM 4-OHT for 4 days or left untreated. The cells were harvested, stained with Annexin V/DAPI and analyzed by flow cytometry on day 4 after the treatment (Day 4), or 3 days later (Day 7). Error bars: SEM. (**C**) β-galactosidase staining was performed in *Rplp1^i^* ^Δ^ and control pMEFs that were treated with 1 µM 4-OHT for 4 days or left untreated. (**D**) Quantification of the amount of β-gal^+^ cells for each analyzed clone analyzed. (**E and F**). Western blot analysis of protein extracts from *Rplp1* ^iΔ^ and control pMEFs that were treated with 1 µM 4-OHT for 4 days or left untreated.

To further understand the role of *Rplp1* in cell proliferation and apoptosis, we generated *Rplp1* ^F/F^ mice after crossing *Rplp1*+/F mice with Cre-ER^T2^ transgenic mice [29], and subsequently isolated the *Rplp1* ^F/F^CER^+^ (*Rplp1* ^iΔ^) and *Rplp1* ^+/F^CER^+^ (control) pMEFs. *Rplp1* was efficiently deleted in the *Rplp1^i^* ^Δ^ pMEFs upon the addition of 1 µM 4-OHT for four days (Figure 7A and 7B). First, we assessed proliferation in *Rplp1^i^* ^Δ^ and control pMEFs that had been treated with 1 µM 4-OHT for four days. *Rplp1* ^iΔ^ pMEFs showed a proliferation defect, with reduced cell numbers (Figure 7C). Furthermore, BrdU labeling of *Rplp1* ^iΔ^ and control pMEFs after four days of 1 µM 4-OHT revealed a considerable reduction of BrdU+ cells among the *Rplp1* ^iΔ^cells, compared with the controls (Figure 7D and 7E). However, when we analyzed the cell cycle profiles, we found no obvious differences in the cell cycle distribution between the control cells and those with a single *Rplp1* allele deletion (Figure 8A). Next, we investigated whether increased apoptosis contributed to the reduced numbers of *Rplp1* ^iΔ^ pMEFs. Fluorescence-activated cell sorting (FACS) analysis revealed no obvious differences in the Annexin V+ cell populations of mutant and control cells that had been stained with Annexin V/DAPI (Figure 8B).

From the growth curve (Figure 7C), we noticed a premature cessation of proliferation in *Rplp1* ^iΔ^ pMEFs after passage 4. To follow the fates of these cells, we performed β-galactosidase (β-gal) staining and found that the frequency of β-gal^+^ cells was higher among *Rplp1* ^iΔ^ pMEFs than among the controls (Figure 8C and 8D), indicating that *Rplp1* deletion caused early senescence in the pMEFs. To investigate the cause of this senescence, we analyzed the levels of important cell cycle regulatory proteins and found higher levels of the CDK inhibitor p16^INK4A^, a marker of senescence associated with the downregulation of pRb activity (Figure 8E). However, the protein levels of p21^CIP1^ and p27^KIP1^ did not differ between the control and *Rplp1* ^iΔ^ pMEFs (Figure 8F). Because senescence can be caused by the accumulation of reactive oxygen species (ROS) [30], [31] and the non-essential ribosomal proteins RPL1, RPL32 and RPL36 can prevent intracellular ROS generation [32], we measured the intracellular ROS levels. No differences were observed between the deleted and control cells (Figure S2), suggesting that ROS was not the cause of senescence in *Rplp1* ^iΔ^ pMEFs. Finally we expressed lentiviral vectors expressing p16^INK4A-shRNA^ and p53^shRNA^ in *Rplp1* ^iΔ^ pMEFs which were further depleted for Rplp1 (see materials and methods). The inhibition of p16^INK4A^ in this context rescues, at least partially, the senescent phenotype while p53 inhibition does not seen to have any effect (data not shown). Moreover transient transfection strategy using siRNAs showed similar results (Figure S3). Taken together, these experiments showed that *Rplp1* deletion in pMEFs induced a p16^INK4A^/pRb pathway-mediated proliferation defect and led to increased senescence.

### *Rplp1* deletion in MEFs does not affect global protein synthesis but alters the translation pattern

**Figure 9:**
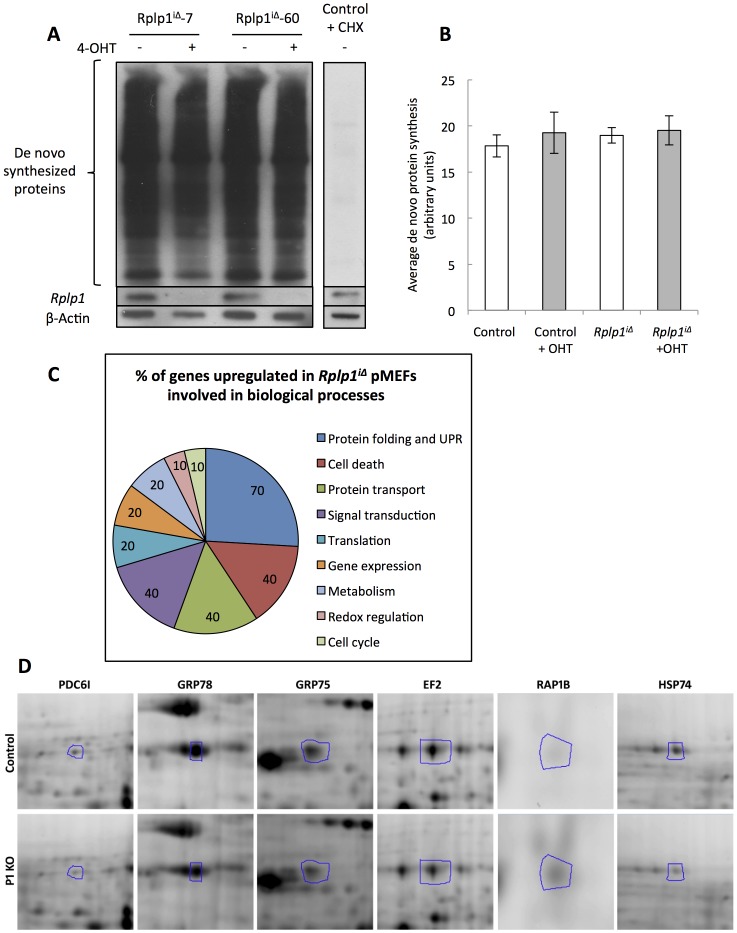
De novo protein synthesis is normal in *Rplp1* ^iΔ^ MEFs, but the protein expression pattern is altered. (**A**) Western blot analysis of newly synthesized proteins in *Rplp1^i^* ^Δ^ MEFs. Cycloheximide (CHX)-treated MEFs were used as a negative control. (**B**) Densitometric quantification of the newly synthesized proteins in *Rplp1* ^iΔ^ MEFs. Two control and 2 *Rplp1^i^* ^Δ^ MEF cell lines were used. Error bars: SEM. (**C**) Percentages of upregulated and downregulated proteins involved in different biological processes in *Rplp1^iΔ^*pMEFs. The classification was performed according to gene ontology (www.geneontology.org). (**D**) Associated alterations in the levels of proteins that are dysregulated by a factor of 1.5 or more in ***Rplp1^i^*** ^Δ^ **pMEFs** cells. Representative photographs of the 2D gel are shown. Only portions of the 2D gel images corresponding to the indicated proteins are shown.

*Rplp1* is important for proper ribosomal interactions with elongation factors, especially eEF-2 [33], [34]. To assess whether Rplp1 deletion affected protein synthesis, *Rplp1* ^iΔ^ and control immortalized MEFs were treated with 4-OHT for four days. Three days after the end of the treatment, the cells were labeled with a methionine analog (azide-homoalanine), and the de novo synthesized proteins were detected by western blotting (Figure 9A) and quantified by densitometry (Figure 9B). Proper *Rplp1* deletion was assessed by western blotting (Figure 9A). The experiment was performed with two pairs of control and *Rplp1^iΔ^* immortalized MEFs. The protein synthesis level was unchanged when *Rplp1* was deleted. Therefore, *Rplp1* deletion in MEFs did not affect general protein synthesis.

Two-dimensional gel electrophoresis was performed to examine the protein translation pattern in *Rplp1*-deficient pMEFs. The proteins that were more strongly dysregulated in *Rplp1* ^iΔ^ pMEFs were selected and identified by mass spectrometry. We identified fourteen proteins that were significantly dysregulated in *Rplp1* ^iΔ^ pMEFs (p<0.01); these were associated with different cellular functions such as protein folding (70%), cell death (40%), gene expression (20%) and metabolism (20%) (Figure 9C and Table S2). In agreement with the finding that protein folding was the most affected group, the upregulation of stress-related proteins such as the endoplasmic reticulum (ER) chaperones HSP74 and GRP78 was confirmed by western blot analysis (Figure 8E).

## Discussion

To study the function of P1 *in vivo*, we generated *Rplp1* knockout mouse models. *Rplp1* null mice could not be generated due to the infertility of *Rplp1^Het^* mice. Surprisingly, *Rplp1^et^* mice, which carry only one *Rplp1* allele, presented with a severe phenotype that led to early postnatal death in approximately 50% of the offspring. *Rplp1^Het^* mice exhibited rigid, kinky, short tails. This phenotype is consistent with the general notion that RP mutational haploinsufficiency is often associated with severe defects in mammals. For example, the phenotype of *Rplp1^Het^* mice is reminiscent of that of Ts (Ts/+) mutant mice, which possess a spontaneous, dominant mutation in *Rpl38*. [1]. While homozygous Ts mutants die before implantation, the heterozygous mice present with a short, kinky tail and skeletal patterning defects [5]. *Rpl38* regulates 80S-mRNA complex formation for specific Hox mRNAs [1]. *Rpl24* heterozygosity in *Bst/+* mice was also shown to cause kinked tails [9]. RPL24 regulates ribosomal subunit association and protein translation [2]. These observations suggest that defects in ribosomal subunit joining or assembly might induce skeletal defects. For other RPs, single-allele deletion leads to lethality during gastrulation, a period during which cell division and differentiation increase drastically, due to an error in ribosomal biogenesis (i. e.: *RpS6* mutations) [2]. In contrast, heterozygous mutations in other RPs such as *Rps19* [35], *Rpl29* [36] and *Rpl24* [9] are compatible with embryonic development. Moreover, some human patients who carry *RPS19* mutations present with skeletal malformations [37].

Our results show that the specific knockout of *Rplp1* in the CNS provoked perinatal lethality and morphological brain defects. The observed brain atrophy was caused by the impaired proliferation and increased apoptosis of neuroprogenitors. *Rplp1* ^CNSΔ^ mice exhibited neocortical apoptosis, which also contributed to brain atrophy. Consistently, deficiencies in other RPs such as *Rpl22* were shown to selectively arrest the development of α/β-T cells in a p53 activation and apoptosis-mediated manner [38]. RPL11 silencing was shown to induce p53-dependent apoptosis [39], and *Rps19* deficiency impaired ribosomal biogenesis and activated p53 in zebrafish [40]. Upon the silencing of other RPs such as RPL23 [41], RPS9 [42] or RPS6 [43], p53 becomes activated and induces apoptosis. In contrast, *Rplp1* deletion led to apoptosis independently of P53. This proliferation defect was accompanied by the downregulation of cyclin E, but appeared to be independent of p16^INK4A^ and p19^ARF^. Surprisingly, the CDK inhibitors p21^CIP1^ and p27^KIP1^ were also downregulated. Although p21^CIP1^ and p27^KIP1^ exert direct cell cycle control at a nuclear level, these proteins can also exert other independent functions when cytoplasmically localized which could contribute to the observed abnormalities of the IZ and CP layers in the *Rplp1* ^CNSΔ^ embryonic brain [44]–[46]. Moreover, the detrimental effect of a single-allele deficiency of *Rplp1* on neurosphere formation might be associated with the downregulation of p21^CIP1^ and p27^KIP1^, as they have previously been associated with cell differentiation and apoptosis [27], [28], [44].

In agreement with our *in vivo* observations, the proliferation defects were also observed in *Rplp1^i^* ^Δ^ pMEFs. Interestingly, the proliferation defect in *Rplp1^i^* ^Δ^ pMEFs induced p16^INK4A^/pRb pathway-mediated premature senescence, which was also p53-independent. Importantly p16^INK4A^ inhibition is able to bypass, at least partially, the senescence phenotype indicating that this pathway is crucial for Rplp1-deficiency-mediated senescence. This finding was consistent with finding that *Rplp1* overexpression could bypass senescence in pMEFs [22]. A similar proliferative defect was also previously observed in P1–P2 knocked-down human cells [47]. In contrast to the *Rplp1^CNS^* ^Δ^ embryonic brain, the p27^KIP1^, p21^CIP1^ and cyclin E protein levels were unaltered in *Rplp1^i^* ^Δ^ pMEFs, indicating that *Rplp1* deletion has different consequences in specific tissues or cell types. This finding was also supported by the fact that, in strong contrast to proliferating cells, *Rplp1* deletion in post-mitotic mature B cells and follicular dendritic cells in *Rplp1* ^BΔ^ mice did not result in an obvious phenotype (Perucho et al., unpublished observations). Given that embryonic stem (ES) cells contain high levels of P1 ([22] and data not shown), our results suggest that *Rplp1* is crucial in embryonic stem cells and progenitor cells (e.g., neural system), but dispensable in certain differentiated cells such as mature B cells. Consistent with this assumption, *Rplp1* expression was downregulated upon the differentiation of mouse ES cells to embryonic bodies [48]. It is also possible that in different cell types, the ability of the ribosomal subunits to compensate for losses in P1/P2 loss differs. For example, P0, P1 and P2 contain a common C-terminal domain that is responsible for the ribosomal interaction with EF-2 [33]. However, the C-terminal domain of P0 is not required for ribosomal activity when P1 and P2 are bound to the ribosome [49]. In other words, the C-terminal domain of P0 might be sufficient for ribosomal-translation factor interactions in some cell types or conditions, whereas in other cell types that require proliferation, P1 and P2 act as rate-limiting factors. For example, the proportions of ribosomal stalk proteins in the cytoplasm have been reported to vary from less than 1% [50] to 75% [51], possibly reflecting the different requirements among organisms and metabolic cell conditions [52].

On the other hand, *Rplp1* deletion (and concurrently *Rplp2* deletion) in the *Rplp1^i^* ^Δ^ pMEFs did not affect global protein translation. This is in agreement with previous observations in human cells [47]. In contrast, the depletion of other RPs (i.e. *Rps9*) was shown to reduce the protein synthesis rate [42]. Interestingly, we identified 14 specific proteins that were dysregulated in *Rplp1^i^* ^Δ^ pMEFs, most of which were ER chaperones. For example, most of the upregulated proteins in *Rplp1^i^* ^Δ^ pMEFs are so in reaction with the ribosomal stress response (i.e.: GRP75, GRP78, SYAC and HSP74). In addition, some of the alterations observed in proliferation and apoptosis could be due to the reduced levels of Rab1b given its role in the control of cell growth [53]–[55]. The ER is home to an array of interlinked chaperone proteins upon which secreted proteins depend for correct folding, partner chain assimilation and final multimer assembly. The ER stress response constitutes a cellular process that is triggered by various conditions that disturb protein folding. Eukaryotic cells have developed an adaptive mechanism, the unfolded protein response (UPR), that acts to remove unfolded proteins and restore ER homeostasis [56]. In mammalian cells, imbalanced RP levels or the absence or malfunction of any RP has been shown to induce ribosomal stress, with severe consequences for cell survival [57]. Although this response has been linked to p53 [58], p53-independent mechanisms are also involved [59], [60]. Interestingly, RP knockdown or haploinsufficiency can provoke a ribosomal stress response that can be associated with senescence, UPR or other mechanisms [61], [62]. The data presented herein link the ER-UPR with the senescence response in a single effector pathway downstream of ribosomal stress. Moreover, because protein folding is coupled with elongation, the absence of P1 and P2 might affect translation elongation by disrupting protein folding. EF-2 upregulation might reflect an attempt by the cell to compensate for the impaired stalk recognition, as P0, P1 and P2 comprise the EF-2 recognition motif [34]. For example, we cannot rule out the possibility that *Rplp1* has additional extra-ribosomal functions, as P2 [63], at replication forks [64]. The relationship between the dysregulated proteins in *Rplp1^i^* ^Δ^ pMEFs and the observed phenotype in the mutant mice remains to be fully characterized. Although the global protein level is unchanged, the absence of P1 might cause an increased translation error rate that would generate unfolded proteins.

Our results suggest that P1 has potential extraribosomal functions related to protein folding, a process that occurs outside the ribosome but within the ER compartment.

On the other hand, it has been hypothesized that *Rplp1* might be involved in internal ribosomal entry site (IRES)-dependent translation [16]. Interestingly, both the p27^KIP1^ and p53 mRNAs contain IRES [65]. This alternative hypothesis should be explored in future studies. Importantly, the P1 disturbance particularly affected proliferating cells (neuroprecursors), strongly suggesting the following: that P1 plays specific roles in the ribosome by “fine-tuning” translation accordingly to the cellular requirements and/or that proliferating cells are equipped with an extra-sensitive ER stress response to preserve their stemness properties. Our work also suggests that *Rplp1* impairment-mediated activation of the ER stress pathway translates into different response mechanisms (senescence *in vitro* and apoptosis *in vivo*) that are incompatible with cell survival and normal development. Overall, our work shows that Rplp1 is crucial for development and proliferation and proposes P1 as a novel factor of protein proper folding and translational “fine-tuning”.

## Supporting Information

Figure S1 H&E staining of *Rplp1* ^Het^ and *Rplp1* ^+/+^ mice tissues. Scale bar: 50 µm. Liver, thymus and brown fat staining were performed at postnatal day 1 (P1). Testis staining was performed at P30.
(TIF)

Figure S2 Senescence is not caused by increased ROS in *Rplp1^i^* ^Δ^pMEFs. An intracellular ROS assay was performed in *Rplp1^i^* ^Δ^and control pMEFs that were treated with 1 µM 4-OHT for 4 days or left untreated.
(TIF)

Figure S3 Senescence is bypassed at least partially by p16^INK4^ inhibition. (**A**) Western-Blot of p16^NK4A^ antibody indicating the efficiency of the p16^INK4AsiRNA^. (**B**) Quantification of senescent cells upon transient transfection with the indicated siRNAs (*p<0.05). (**C**) Photographs of *Rplp1^i^* ^Δ^ and control pMEFs transfected with the p16^INK4AsiRNA^ and further treated during 4 days with 1 M 4-OHT.
(TIFF)

Table S1 List of antibodies used.
(DOC)

Table S2 Dysregulated proteins identified in *Rplp1^i^* ^Δ^ pMEFs by two-dimensional gel electrophoresis and classification according to gene ontology (www.geneontology.org) in the different biological processes in which they are involved. Green indicates protein involvement in a biological process and red indicates non-involvement.
(XLSX)
