# Supplementary figures and images for: RPLP1, a Crucial Ribosomal Protein for Embryonic Development of the Nervous System
Source: PLoS One. 2014 Jun 24;9(6):e99956. doi: 10.1371/journal.pone.0099956 (PMC4069005; doi:10.1371/journal.pone.0099956)

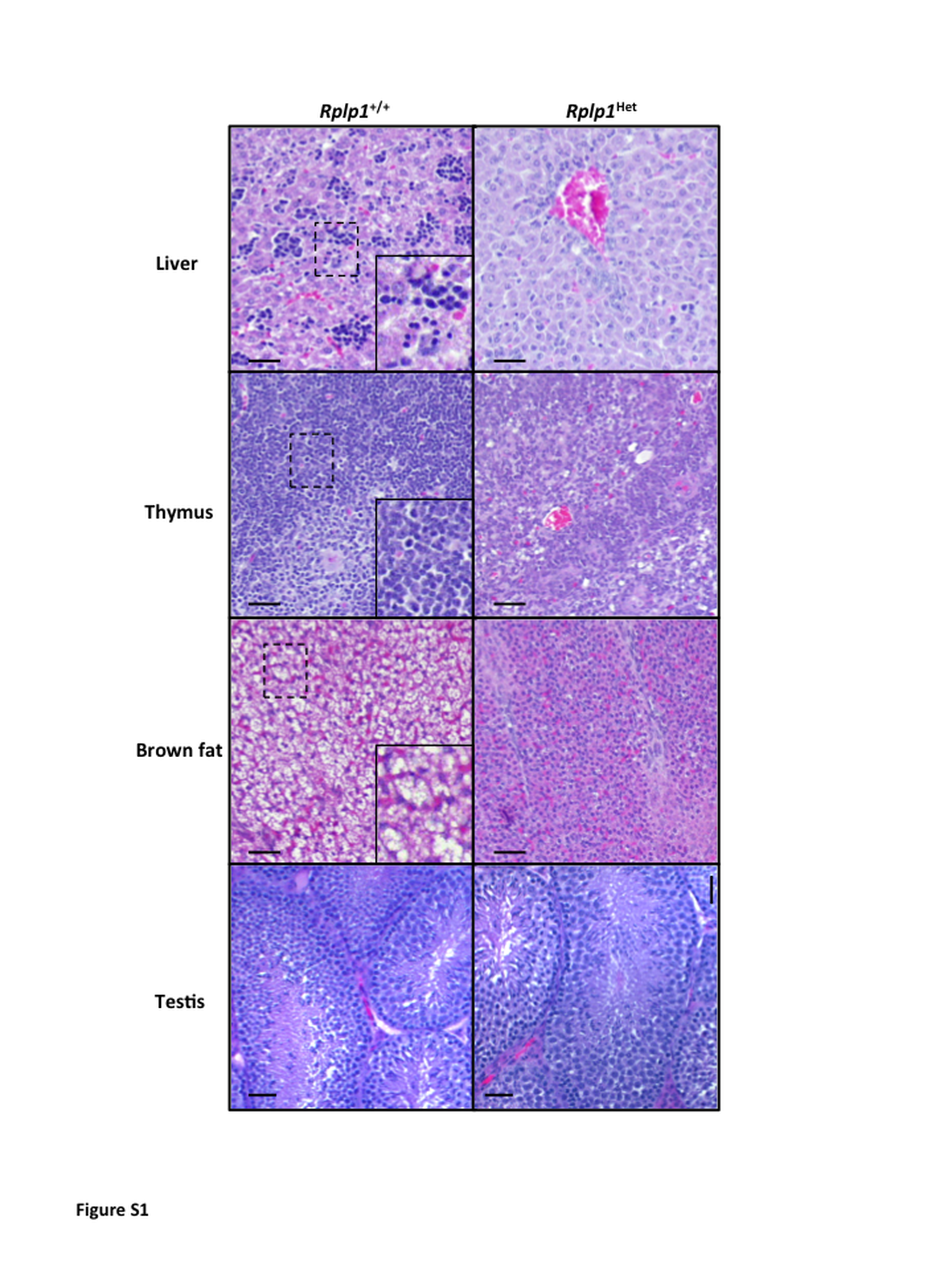

Supplement: Figure S1 — H&E staining of Rplp1 Het and Rplp1 +/+ mice tissues. Scale bar: 50 µm. Liver, thymus and brown fat staining were performed at postnatal day 1 (P1). Testis staining was performed at P30. (TIF) [file pone.0099956.s001.tif]

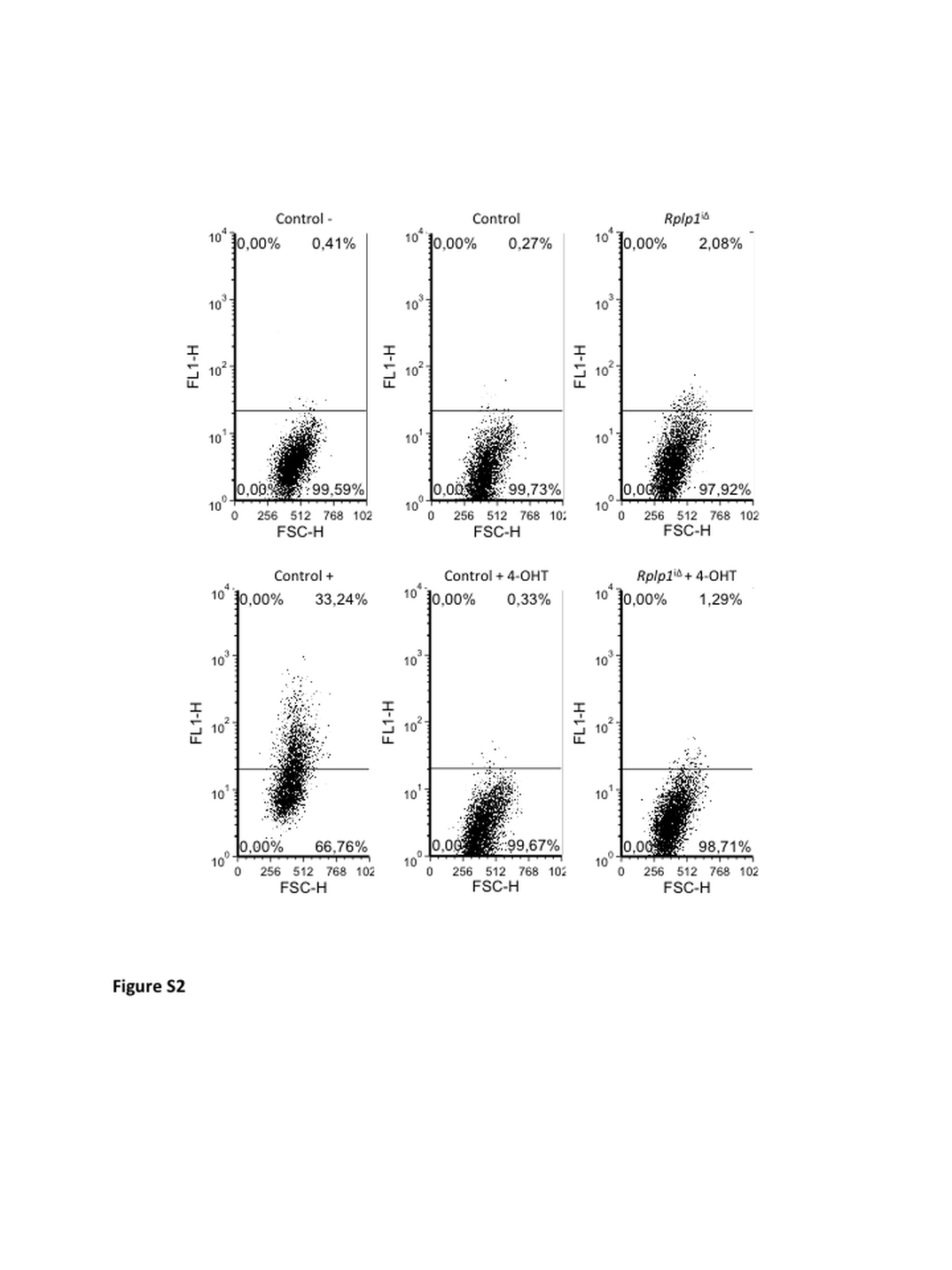

Supplement: Figure S2 — Senescence is not caused by increased ROS in Rplp1i ΔpMEFs. An intracellular ROS assay was performed in Rplp1i Δand control pMEFs that were treated with 1 µM 4-OHT for 4 days or left untreated. (TIF) [file pone.0099956.s002.tif]

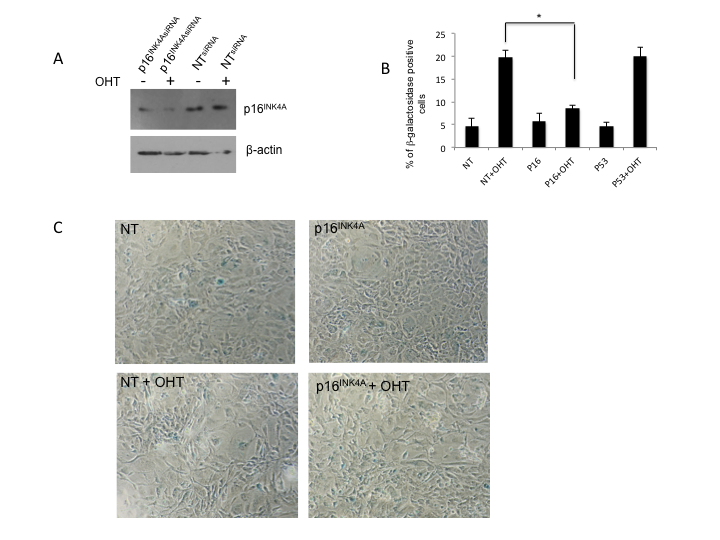

Supplement: Figure S3 — Senescence is bypassed at least partially by p16INK4 inhibition. (A) Western-Blot of p16NK4A antibody indicating the efficiency of the p16INK4AsiRNA. (B) Quantification of senescent cells upon transient transfection with the indicated siRNAs (*p<0.05). (C) Photographs of Rplp1i Δ and control pMEFs transfected with the p16INK4AsiRNA and further treated during 4 days with 1 M 4-OHT. (TIFF) [file pone.0099956.s003.tiff]
