# Supplementary material for: RPLP1, a Crucial Ribosomal Protein for Embryonic Development of the Nervous System
Source: PLoS One. 2014 Jun 24;9(6):e99956. doi: 10.1371/journal.pone.0099956 (PMC4069005; doi:10.1371/journal.pone.0099956)
Supplement: Table S1 — List of antibodies used. (DOC) [file pone.0099956.s004.doc]

**Table S1: Antibodies used**

Primary antibodies

| **Antigen** | **Species** | **Company** |
| --- | --- | --- |
| Ki67 | Rabbit polyclonal | Neomarkers |
| BrdU (clone BU1/75 [ICR1]) | Rat monoclonal | Abcam |
| P27/Kip1 | Mouse polyclonal | BD Bioscience |
| P21 | Rabbit polyclonal | Abcam |
| P53 | Sheep polyclonal | Merck |
| P16 (M-156) | Rabbit polyclonal | Santa Cruz |
| CyclinA | Rabbit polyclonal | Santa cruz |
| CyclinE | Rabbit polyclonal | Santa Cruz |
| Rplp1 | Rabbit polyclonal | Sigma |
| Rplps | Rabbit polyclonal | Homemade (Vilella*et al*., 1991) |
| Tuj1 | Mouse polyclonal | Covance |
| pH3 (phospho-S10 Histone 3) | Rabbit polyclonal | Millipore |
| β-Actin | Mouse polyclonal | Sigma-Aldrich |
| β -Tubulin | Mouse polyclonal | Sigma-Aldrich |
| Caspase-3 (cleaved) | Rabbit polyclonal | Cell Signalling |
| CDKN2A/p19^ARF^ | Rabbit polyclonal | Abcam |
| E2F1 | Rabbit polyclonal | Santa Cruz |
| HSPA4/Apg-2 | Rabbit polyclonal | Cell Signalling |
| GRP78/BiP (GL-19) | Rabbit polyclonal | Sigma Aldrich |
| P53 (FL-393) | Rabbit polyclonal | Santa Cruz |
| Rb | Rabbit polyclonal | BD Bioscience |
| Cd11b-biotin | Mouse monoclonal | eBiosciences |
| B220-PerCpCy5.5 | Rat monoclonal | eBiosciences |
| CD19-PECy7 | Rat monoclonal | eBiosciences |
| CD23-FITC | Rat monoclonal | eBiosciences |
| CD21-PE | Mouse monoclonal | eBiosciences |

Secondary antibodies

| **Antigen** | **Species** | **Conjugate** | **Company** |
| --- | --- | --- | --- |
| Mouse IgG F(ab’)_2_ | Sheep | FITC | Sigma-Aldrich |
| Mouse IgG F(ab’)_2_ | Sheep | Cy3 | Sigma-Aldrich |
| Rabbit IgG F(ab’)_2_ | Sheep | Cy3 | Sigma-Aldrich |
| Rabbit IgG F(ab’)_2_ | Goat | FITC | Sigma-Aldrich |
| Rat IgG F(ab’)_2_ | Goat | Cy2 | Jackson ImmunoResearch |
| Mouse whole IgG | Goat | HRP | Dako |
| Rabbit whole IgG | Goat | HRP | Dako |
| Sheep whole IgG | Rabbit | HRP | Dako |
| Goat whole IgG | Rabbit | HRP | Dako |

IHC:Immunohistochemistry. WB: Western blot. FC: Flow cytometry. PerCp: PeridininChlorophyl Protein Complex. Cy: cyanine. PE: Phycoerythrin. FITC: Fluorescein isothiocyanate. HRP: Horseradish peroxidase. IgG: Immunoglobulin G. F(ab')_2_: Fragment antigen-binding.
